# Response to diagnosis of pre-diabetes in socioeconomically deprived areas: a qualitative study

**DOI:** 10.3399/bjgpopen19X101661

**Published:** 2019-09-04

**Authors:** Helen Twohig, Victoria Hodges, Chloe Hobbis, Caroline Mitchell

**Affiliations:** 1 Wellcome Trust Primary Care Doctoral Fellow, Primary Care Centre Versus Arthritis, School of Primary, Community and Social Care, Keele University, Keele, UK; 2 Clinical Fellow, Academic Department of Primary Medical Care, University of Sheffield, Sheffield, UK; 3 Academic Clinical Fellow, Academic Department of Primary Medical Care, University of Sheffield, Sheffield, UK; 4 Medical Student, Academic Department of Primary Medical Care, University of Sheffield, Sheffield, UK; 5 Senior Clinical Lecturer, Academic Department of Primary Medical Care, University of Sheffield, Sheffield, UK

**Keywords:** general practice, diabetes mellitus, pre-diabetes, socioeconomic deprivation, behaviour change, life style

## Abstract

**Background:**

Diabetes prevention is a key priority for the NHS, with a particular focus on populations at highest risk. The NHS Diabetes Prevention Programme (NHS DPP) has been introduced, offering a course of dietary and lifestyle education to individuals with pre-diabetes. However, concerns about the NHS DPP include: (1) the possible unintended consequences of labelling more people with a ‘pre-condition’; (2) the possibility of worsening health inequalities as people in socioeconomically deprived areas tend to access behaviour-change programmes less readily; (3) the appropriateness of an intervention focused on individuals versus population-wide public health policy interventions.

**Aim:**

To explore the experience of diagnosis of pre-diabetes, and understand the barriers and facilitators to uptake of the NHS DPP for people living in socioeconomically deprived areas.

**Design & setting:**

A qualitative study was undertaken. Participants with pre-diabetes were recruited from practices serving socioeconomically deprived areas of Sheffield, UK.

**Method:**

Semi-structured interviews were conducted and continued until data saturation (23 participants). Thematic analysis of data was undertaken.

**Results:**

Both healthcare context and an individual’s personal and community context shaped response to diagnosis and likelihood of engaging with the NHS DPP. Patient activation was a useful concept in understanding response. Whether or not people participated in the NHS DPP, being diagnosed with pre-diabetes tended to provoke some degree of dietary change and did not cause significant anxiety for most. However, there were multiple barriers to engaging with the NHS DPP for this patient group.

**Conclusion:**

Diagnosing pre-diabetes can provoke an individual positive response, but the sociocultural environment often limits an individual’s ability to engage with the NHS DPP or make lifestyle change.

## How this fits in

People living in socioeconomically deprived areas are at higher risk of developing diabetes than those in better-off areas, but may be less able to access lifestyle intervention programmes. This is the first study to explore the experience of diagnosis of pre-diabetes for people living in socioeconomically deprived areas since the recent introduction of the NHS DPP. It highlights the complex interplay between health care and a person’s sociocultural environment in shaping response to the diagnosis and decision to engage or not with the NHS DPP.

## Introduction

It is estimated that 30% of the adult UK population is pre-diabetic.^1^ Of these, 5%–10% will progress to type two diabetes mellitus each year, while a similar proportion will return to normoglycaemia. Ten per cent of the NHS budget is spent on diabetes care.^2^ With rates of diabetes set to rise further, diabetes prevention has become a key priority for the NHS.^3^

Identifying individuals with pre-diabetes and intervening to try to reduce their risk of progression to type two diabetes is one strategy being employed. A diabetes prevention programme (the NHS DPP) has recently been introduced across England.^4^ This 9-month individualised lifestyle intervention programme aims to reduce progression to type two diabetes through education on healthy eating and lifestyle, support with weight loss if appropriate, and bespoke physical exercise programmes.

A randomised controlled trial of a smaller scale intervention programme in the UK (‘Let's Prevent’) found that patients from socioeconomically deprived groups were less likely to take up offers of lifestyle interventions and less likely to remain engaged with them than the general population.^5^ Diabetes and its associated complications disproportionately affect certain minority ethnic groups and those in socioeconomically deprived populations.^6^ A failure to reach these high-risk groups with a nationally funded prevention programme, therefore, has the potential to widen health inequalities.

A recent meta-narrative review by Barry *et al* ^7^ cautions that the biomedical paradigm (which views pre-diabetes as a reversible state of abnormal glucose metabolism that can be corrected by altering an individual patient's lifestyle) leads to policy that neglects to consider the complex sociocultural environment in which people live.

An additional concern is that pre-diabetes is an arbitrarily defined condition along a spectrum of the range of HbA1c, and the introduction of this category has expanded the range over which HbA1c is considered ‘abnormal’. This has potential to medicalise people who will never develop symptoms or complications from the condition and has opportunity costs in an already overstretched primary care system.

The aim of the study was to explore the experience of diagnosis of pre-diabetes for people living in socioeconomically deprived areas, in order to gain insight into the interplay between people’s sociocultural environment, their understanding of, and response to, the diagnosis, and their decision to engage or not with the NHS DPP.

## Method

### Design

A scoping literature review informed development of the interview topic guide, which was discussed with the ‘Deep End Yorkshire Humber’ (DEYH)^8^ patient and public involvement (PPI) group. DEYH, established in 2015 and inspired by the Glasgow ‘GPs at the Deep End’ group,^9^ brings together general practice-based healthcare professionals working in the most socioeconomically deprived areas of the region and has links with academia, and the voluntary and social care sectors.

### Participant identification and recruitment

Participants were primarily recruited through the DEYH National Institute of Health Research Yorkshire and Humber Clinical Research Network cluster, a group of research-active practices that are among the 100 most socioeconomically deprived practice areas of the whole region. Purposive sampling was used to achieve a range of ages, sex, and ethnic groups.

People were eligible if they were aged >18 years, had a coded diagnosis of pre-diabetes assigned within the preceeding 12 months (by HbA1c test result of 42–47 mmol/mol), and had been offered referral to the NHS DPP. Exclusion criteria were poor prognosis from any comorbidity such that participation in the study was not appropriate, or a level of English that was insufficient to participate in an interview.

Potential participants were sent a copy of a patient information sheet and a consent form with a covering letter from their practice. Those who returned the reply slip to the research team were contacted to arrange an interview. Participants were compensated for their time with a £15 shopping voucher.

### Data collection

Individual semi-structured interviews were conducted (see Box 1 for sample questions from the topic guide). In some cases, a relative was present and contributed to the discussion. Data analysis was undertaken concurrently with the topic guide, adjusted for subsequent interviews to ensure emerging themes were explored.

Box 1 **Interview topic guide example questions**

**Table:** 

Section 1Introduction	Explanation of the study and the interview processConsentCheck demographic details
Section 2Participant’s story	Can you tell me about when you first heard your blood sugar result was abnormal?Do you recall a discussion about referral to the Healthier You: NHS Diabetes Prevention Programme?
Section 3Understanding of pre-diabetes	What do you understand about pre-diabetes?How did you feel about being told you had it and/or had a raised blood sugar?Did you discuss it with family and friends?Have you changed anything in your lifestyle since?
Section 4Perception of the DPP before attending	Why did you or didn’t you accept referral to the programme?What did you expect from the programme?How did being offered referral make you feel?
Section 5Experience of the DPP if they have attended	How has the programme been for you?What was good or bad about it?Have you made any changes as a result of going?
Section 6Close	Anything else?Summarise and conclude

DPP = diabetes prevention programme.

Interviews were conducted by an academic GP or a medical student, both of whom had received training in interview skills for qualitative research. Interviews were audio-recorded and transcribed verbatim. Transcripts were checked for accuracy and anonymised.

### Analysis

A constructivist epistemological approach was taken. Constructivism considers the interplay between the subject and the world around them.^10^ It also accounts for the influences that culture and social surroundings have on an individual. Data were analysed inductively using thematic analysis^11^ with an interpretivist analytical approach.^12^ Two researchers repeatedly read through the transcripts to immerse themselves in the data. Initial codes were generated and organised into themes using NVIVO (version 11) software. Interviews continued until data saturation was evident in a maximum variety sample of participants. A series of meetings with the full research team were held, during which themes were debated and refined to confirm data saturation and inform the final conceptual framework.

## Results

### Participants

A total of 23 people with pre-diabetes were interviewed, 13 female and 10 male. Mean index of multiple deprivation (IMD) score was 43.7 and mean age was 61 years (range 37–81 years). Sixty-nine per cent of participants were white British, 17% Asian, 9% African, and 5% Carribean. The majority of participants had a family history of type two diabetes (*n* = 13/23). Three were attending the NHS DPP; three had started the programme but dropped out; eight had accepted referral and were waiting for an appointment; four had declined referral; and five did not recall being offered referral. See Table 1 for summary participant data.

**Table 1.** Demographic data of participants

ID	Age, years	Sex	Ethnic group	Employment	Family history	IMD quintile	DPP referral status
P1	45–50	M	African	Unemployed	Yes	5	No referral offered
P2	50–55	F	White British	Employed	No	5	No referral offered
P3	50–54	M	White British	Unemployed	Yes	5	Attending the DPP
P4	55–59	F	Pakistani	Retired	Yes	5	Attending the DPP
P5	50–54	F	White British	Unemployed	Yes	5	Referred, waiting to hear
P6	40–44	M	Pakistani	Employed	No	5	Referred, waiting to hear
P7	40–44	F	Pakistani	Unemployed	Yes	5	Attended but stopped going
P8	70–74	F	White British	Retired	No	5	Referred, waiting to hear
P9	75–79	M	White British	Retired	No	4	Referred, waiting to hear
P10	75–79	M	White British	Retired	No	4	No referral offered
P11	30–34	M	Asian	Employed	Yes	5	Declined
P12	65–69	F	White British	Retired	No	4	Referred, waiting to hear
P13	80–84	M	White British	Retired	No	5	Attended but stopped going
P14	70–74	F	White British	Retired	Yes	4	Declined
P15	55–59	M	White British	Retired	Yes	5	Declined
P16	45–49	F	African	Retired	Yes	5	Referred, waiting to hear
P17	60–64	F	White British	Retired	Yes	4	No referral offered
P18	55–59	F	White British	Unemployed	Yes	5	Declined
P19	50–54	F	Caribbean	Unemployed	No	5	Referred, waiting to hear
P20	80–85	F	White British	Retired	No	4	Attended but stopped going
P21	70–74	F	White British	Retired	Yes	4	No referral offered
P22	70–74	M	White British	Retired	No	4	Attending the DPP
P23	65–69	M	White British	Retired	Yes	3	Referred, waiting to hear

ID = identifier. IMD = index multiple deprivation. DPP = diabetes prevention programme.

### Themes

Five main themes were identified that described participants’ understanding of, and response to, the diagnosis and their decision to engage with the NHS DPP: (1) the healthcare context; (2) personal and community context; (3) patient activation; (4) acceptance; and (5) diagnosis as a motivator.

#### 1. Healthcare context

The healthcare context, in relation to both testing and information giving, shaped interviewees’ responses to the diagnosis.

##### Route to testing

In most cases, participants were not explicitly aware that they were having their HbA1c levels checked; for example, if it was part of a review of other long-term conditions. This meant that they were not expecting the results when they were delivered:

*‘So our concern was actually his gout problems, so we went to the GP to have his blood taken to check on his acid uric level, but apparently at the same time other things were also checked from his blood sample and one of his is to measure his sugar level.’* (P11)

For the majority of participants, the unexpected information was presented positively and framed to emphasise that knowledge of the condition meant that action could be taken early before diabetes developed:

*‘It’s good news to know early and know that he still has chance to go back to normal.’* (P11)

In a few cases, however, the unexpected abnormal result caused feelings of vulnerability and ‘illness’:

*‘ … in July they took me bloods. And they're always come back, for't last 5 years fine. And then this year, I got this, this letter that I got this er dangerous, or start of being dangerous, this number 42 on't scale, which shook me, because all at once, never would have considered that I'd got any problems at all and when I say that, I don't know why I say that because I mean, I don't know how to protect myself, or didn't know how to protect myself against getting diabetes.’* (P22)

In situations where people had HbA1c testing when they had comorbidities that they understood to be associated, or where people were experiencing as yet unexplained symptoms, the diagnosis of pre-diabetes was less unexpected, even if these symptoms were misattributed:

*‘That’s when they decided right we’ll take blood tests for anything else that might occur because, in a way, it will be related, you know, a heart condition and diabetes* […] *I was shocked but, in a way, I wasn’t that surprised, I just had that funny feeling something’s not quite right.’* (P3)

##### Healthcare professionals: sceptics or enthusiasts

The healthcare professional delivering the diagnosis played a key role in how patients responded to the diagnosis, influencing participants’ emotional responses, subsequent lifestyle changes, and decision to accept referral. Healthcare professionals could be described as either ‘sceptics’ or ‘enthusiasts’. Enthusiasts presented the diagnosis as an important health priority and urged their patient to attend the NHS DPP, sometimes despite significant reluctance on the patient's part:

*'“Oh, you are on the borderline,” which as I said, I just totally disbelieved, however, I eventually, I was, if you like, pressurised to meet S, I can’t remember her surname, but this S was at the surgery and she came and said, “Look, it’s in your own best interests to attend these meetings”.'* (P20)

This participant attended the programme despite having mobility problems and having to rely on a relative to take her.

Sceptics minimised or dismissed the diagnosis as irrelevant. One participant, who declined referral, described an initial conversation with a nurse who was enthusiastic about the NHS DPP but then a subsequent consultation with their GP (with whom they described having a long-term trusting relationship), which ultimately swayed their decision:

*' … he said, “really, I was on holiday last week, but if I’d have been here and this blood tests came back I wouldn’t have sent for you*”.' (P14)

In most cases, participants decision to attend the programme aligned with the ‘enthusiasm’ or ‘scepticism’ of the healthcare professional. Most who described conversations with ‘sceptical’ healthcare professionals found the minimising approach reassuring. However, for some, a perceived lack of action caused significant worry:

*'So I says so, “Are you going to test it now, to see what it is now? ‘Cause a week’s gone by?” and she says, “No, we'll wait til something happens.” That's the gospel truth. So I just got up and said, “Fair enough then” and walked out … it is a worry really, because I know what can happen with diabetes and since I’ve been going up to’t Northern General to’t rehabilitation centre and seeing’t amputees an’ what have you, it really shocks you dun’t it?*' (P17)

This participant did not recall being offered referral to the NHS DPP.

#### 2. Personal and community context

##### Normalisation of diabetes

Participants’ knowledge of diabetes through experience of diabetes in relatives and friends was a key factor in how they responded to the diagnosis. Normalisation of diabetes within communities meant the impact of being diagnosed with pre-diabetes was lessened:

*‘I know his mum’s got type two so she has to watch what she eats. His uncle is actually in here at the minute, he’s just passing, he’s got diabetes. His dad’s got diabetes.’* (P2)

‘ [diabetes] *it’s just part of everyday life.’* (P5)

However, where people had awareness of family or friends with complications of diabetes, the diagnosis of pre-diabetes caused more concern:

*‘Because I knew my mum died from diabetes so that made me scared, you know, to tell you the truth*.’ (P1)

##### Relationships, roles, and responsibilities

The sociocultural environment of an individual affected their ability to enact lifestyle change and engagement with the NHS DPP. Family could act as either a facilitator, for example providing transport and joining in with lifestyle change at home, or as barrier. Caring responsibilities for children or older or sick relatives were frequently cited as barriers to engagement:

*‘* *I’ve got, like, when the kids go back to school I’ve got commitments helping my mate because her youngest two are twins and the little lad, he’s got autism, so I go and help* *her and I take them home, so it all depends on that.’* (P5)

The central role of food in family life, cultural dietary norms, and the tendency, in this sample of participants, for women in the family to be the main cooks, also affected decisions to attend and the ability to make changes to diet:

*‘I changed the whole family’s* [diet], *because when we’re sitting and we’re cooking, my whole family’s sitting, like, my daughter-in-law, my son, two sons, my husband, we’re sitting, because I cook, you know, my daughter and me cooking.’* (P4)

##### Resource constraints

Difficulty affording and using public transport was a frequently cited barrier to attending the NHS DPP, especially where there were coexisting, painful physical conditions limiting mobility:

*‘It all depends on where it is and like if I've got money to get there because at the moment, my DLA* [Disability Living Allowance]*’s been stopped so I'm on PIP* [Personal Independence Payment]*.’* (P5)

Concerns about personal safety, while travelling to the programme through areas seen as unsafe, were also raised:

*'If I’m stood at the bus and somebody stands behind me I’ll move round to’t side of him and he’ll say, “It’s alright, they’re not doing nowt,” and I’ll say “No, but I don’t like it, you know I don’t like it.” And I think another thing what’s caused it is because there’s all this knife crime and shootings going on and that’s really had an effect.'* (P18)

Eating healthily was also felt to be more expensive:

*‘It’s not always easy to follow these things because sometimes buying certain foods oh I’ll buy that instead of that because it’s got you know low traffic lights you know and then I look a price and oh crikey.’* (P13)

In addition to constrained financial resources, time and personal capability to focus on health were also scarce for many participants, who were overwhelmed by multiple other demands:

*'Sometimes I have no time because I live in joint family — my father-in-law and my mother-in-law and my brother-in-law — that’s very problem, sometimes there is cooking, that’s why I can't go to outside.'* (P6)

#### 3. Patient activation

Patient activation is a term used to describe a person’s knowledge, skills, and confidence to manage their own health.^13^ It became apparent during the analysis that this was a key concept in understanding participants’ attitudes and responses to being told they were pre-diabetic.

Participants’ understanding of the term ‘pre-diabetes’ (knowledge) and their ability to navigate health information and services (skills), influenced how they felt about and reacted to the diagnosis.

Some highly activated participants understood the condition to be a reversible state that they could influence:

*‘ … you’ve been given an early sign that something, diabetes, the first stages of diabetes is going to set in, so obviously got to do something about it. Obviously start making subtle changes to your food and all that and your lifestyle.’* (P3)

Those who appeared to have lower levels of activation saw it as a step towards the inevitable development of type two diabetes:

*‘That eventually I will get diabetes, that’s all they said. It could be a few years but they're going to try and keep at bay as long as they can, you know, but eventually I will be diabetic*.’ (P8)

Some participants passively accepted advice from healthcare professionals and if they were offered referral, were likely to attend the programme:

*‘* *She said* *,* *"* *W* *ell you don’t have to, but if I were you I’d go through it* *,* *"* *so that’s why I did it.’* (P9)

However, others did not have the capacity to even begin to consider preventive healthcare measures owing to the constant pressure of their adverse social circumstances. For example, one participant, who was an unemployed single parent, clearly was not in a position to engage with a letter from his practice about pre-diabetes at that point in his life:

*‘I’m sure I got a letter or something. I can’t remember. I got something, you know, like a — yeah, I think I got a letter about that. Yeah, yeah. I got a letter, I can remember … I just put it on the side. But yeah, yeah, I was going to talk to the doctor about it but I’ve got a lot of things, yeah.’* (P1)

Some participants that did accept referral wanted to attend the programme in the hope of receiving information to guide their lifestyle changes. This mid-level of activation is a pivotal stage where an individual knows they have a role to play in improving their health but needs guidance and support to do so:

*‘Well I'm just hoping, I think I'm eating healthy and everything but I want professional opinions to put me on the right track* *.* *’* (P8)

#### 4. Acceptance

The majority of participants appeared to accept the diagnosis of pre-diabetes without significant associated distress. This finding was closely linked with themes already discussed, including the attitude of the healthcare professional and the sociocultural normalisation of diabetes:

*‘It were just following, like, it runs in the family so I probably knew I were going to come down, get it, so just a case of when and where or where I’ll be* […] *I think it’ll probably happen because, like I said, it runs in the family, so it’s a case of I could get it, I might not get it, but on the borderline means that I probably would get it and I’d be able to deal with it now I know but, yeah, doesn’t bother me.’* (P5)

Acceptance of the diagnosis was not necessarily a feature of having low levels of activation, as some participants still felt they could influence the course of events, but they did not view the diagnosis as a significant, disruptive event in their lives.

There were some disconfirming cases where participants were distressed by being given information about their results (see quotations from P1, P17, P22 above) but these were the minority.

#### 5. Diagnosis as a motivator

Almost all participants, whichever way the diagnosis was presented, described making some positive changes to their lifestyle. For most, this was a dietary change:

*‘I mean, first thing I did was throw the Christmas cake away and I went shopping the day after for bread, different bread, because normally we have white bread, I went out and found a granary type loaf.’* (P12)

However, participants also highlighted barriers to making changes, including competing demands on their time and comorbidities preventing physical activity:

*‘At the moment I’m suffering with severe psoriasis on my feet and every time I walk the skin’s just breaking and it’s so painful to walk and plus I get breathless when I’m walking.’* (P18)

Several participants explained that they had had lifestyle advice on multiple occasions but that an asymptomatic ‘pre-condition’ was an insufficient motivator to make changes:

*‘If I started having any symptoms or I was ill I would go to the doctors and if they had said* *“yes you are on the borderline* *” then I might do something about it.’* (P14)

Other factors cited as motivation to make changes were fear of complications of diabetes and a wish to avoid taking tablets.

## Discussion

### Summary

Participants’ responses to a diagnosis of pre-diabetes was shaped by a combination of the healthcare context and their personal and community context. This included factors such as their path to testing, the attitude of the healthcare professional delivering the information, normalisation of diabetes in communities, personal roles and responsibilities, and resource constraints. In this deprived population, there were individuals whose social circumstances were so overwhelming that the capacity to prioritise health was outweighed by the day-to-day struggle of existence. The theme of patient activation emerged strongly, cutting through the other themes and linking emotional response and subsequent action. In general, the diagnosis did not cause significant anxiety and, even in those who declined referral to the NHS DPP, the information frequently provoked some degree of lifestyle change.

### Strengths and limitations

As the availability of lifestyle intervention programmes for people with pre-diabetes is a recent development in the UK, there are few studies of the effect on individuals of explicitly providing this diagnosis or on reasons for uptake or not of referral to such programmes. This is the first study to explore these issues specifically in people living in areas of significant socioeconomic deprivation, and this is important because of the known disproportionate rates of obesity and diabetes in these communities.

Using the DEYH research cluster, it was possible to recruit people from the most deprived areas of Sheffield; in addition, almost a third of participants were from black and minority ethnic groups, reflecting the demographic who have highest prevalence of type two diabetes. Having funded interpreters for interviews would have increased the diversity of participants. While an interpretivist theoretical approach to analysis was sought (which has been identified as a gap in the literature in this field),^7^ the research team’s inherent bias, as clinicians, towards the biomedical paradigm is recognised. Mitigating this to some degree was the fact that, as well as being GPs, the team members collectively had significant experience in qualitative research and had regular discussions throughout the process to promote reflection.

In some participants, there was a time period of several months between pre-diabetes diagnosis and taking part in the study, which could have influenced recall of events. As only a few participants had actually attended the course by the time of interview, reasons for continued engagement or disengagement were not focused on, but this could be an area for future research.

### Comparison with existing literature

Controversy exists around the validity of the concept of pre-diabetes and other such 'pre-conditions'.^14–16^ The disclosure of similar conditions where the boundary is blurred between ‘risk factor’ and ‘disease’ (for example, early stage chronic kidney disease), is known to provoke anxiety in practitioners who are wary of causing harm to patients by over-medicalisation.^17^ In this study, most participants accepted the diagnosis of pre-diabetes without distress, seeing it as an opportunity to act to reduce the risk of development of diabetes. This suggests fear of causing widespread individual anxiety may be unfounded; although, the potential broader harms of over-diagnosis^18^ still exist.

Despite the normalisation of diabetes, most participants described having made lifestyle changes as a result of being told they were in a high-risk group. This was independent of whether or not they agreed to referral to the NHS DPP. The provision of the diagnosis itself, particularly if information was given about its reversibility, acted as a brief intervention,^19^ appearing to promote some degree of positive lifestyle change in the majority.

This apparent beneficial effect of simply discussing pre-diabetes was unexpected. Studies in the field of behaviour change have tended to find that provision of information about risk is not sufficient to motivate lifestyle change,^20,21^ and previous qualitative work in people with pre-diabetes suggested that the diagnosis caused uncertainty without tangible benefit.^22^ It may be that the existence of a programme for follow-up and support, and the increasing awareness of the term pre-diabetes since the previous qualitative work has countered some of the identified uncertainty. This fits well with the COM-B model of behaviour change in which three essential conditions — Capability (physical and psychological), Opportunity (social and physical), and Motivation (automatic and reflective) — sit at the inner ‘hub’ of a Behaviour change wheel.^23^ For the majority of participants in this study, knowledge that they were at higher risk of diabetes and provision of information about how to reduce this risk acted as motivation and improved capability to make changes, and for some the NHS DPP offered an opportunity to help enable behaviour change (and potentially further enhance capability and motivation).

It is known that only approximately one-third of people identified as being at high risk of diabetes take up an offer of a lifestyle intervention programme, but there has been little exploration of reasons for this.^7,24^ The findings in this current study highlight the complexity of the interplay between intrinsic personal factors and external social and environmental factors when making decisions about attendance at the NHS DPP. A recent study in New Zealand explored barriers and facilitators to dietary change in people with pre-diabetes who had been through a 6-month goal setting and dietary education programme.^25^ The barriers to change identified at the end of the programme were lack of family support, social expectations and pressure around food, financial constraints, and other chronic health problems; that is, despite the intervention, personal, social, and environmental pressures limited people’s ability to make lifestyle changes.

One striking finding in the present study was the strength of influence of the initial consultation with the healthcare professional. There have been recent studies that have explored attitudes to the concept of pre-diabetes from a healthcare professional perspective. Burch *et al* found that UK primary care practitioners adjusted what they discussed with a patient and whether a referral to the NHS DPP was offered according to the age of, and perceived risk or benefit to, the patient.^26^ Mainous *et al* surveyed family physicians in the US and found that those who had a positive attitude to pre-diabetes as a clinical construct were more likely to follow screening and management guidelines.^27^ Findings from the present study reflect this from a patient perspective. Many participants reported a rather paternalistic consultation in which the diagnosis was either minimised or portrayed as a significant threat to future health. Given the ongoing debate about the best approach to diabetes prevention, it is not surprising that professional attitudes vary but there was little evidence in this small sample of shared decision-making practices that might have better served the patients.

### Implications for research and practice

For participants in this study, information about their higher risk of diabetes provided motivation to make lifestyle changes. However, the multitude of barriers to engaging with the NHS DPP that exist for this patient group, along with the acceptance that diabetes is an expected part of life for many, emphasises that an individualised lifestyle intervention in the absence of change to the sociocultural environment will only ever go a small way to effecting change in population rates of type two diabetes.

Many patients ‘at risk’ of diabetes in socioeconomically deprived areas welcome the opportunity for diabetes prevention and are willing to engage in lifestyle change. The point of diagnosis of pre-diabetes may be a powerful ‘teachable moment’. However, consideration should be given to more judicious and explicit testing, along with acknowledgement of practitioners’ own bias when discussing results.

While there are patients who require or desire intensive guidance for lifestyle change, this is not appropriate for all. Further research to develop a primary care-delivered brief intervention to use with patients at the point of diagnosis with pre-diabetes could fill this gap.
